# Metal Oxide Gas Sensor Drift Compensation Using a Dynamic Classifier Ensemble Based on Fitting

**DOI:** 10.3390/sl30709160

**Published:** 2013-07-17

**Authors:** Hang Liu, Zhenan Tang

**Affiliations:** College of Electronic Science and Technology, Dalian University of Technology, No.2 Linggong Road, Dalian 116024, China; E-Mail: tangza@dlut.edu.cn

**Keywords:** sensor drift, metal oxide sensors, ensemble method, dynamic weights

## Abstract

Sensor drift is currently the most challenging problem in gas sensing. We propose a novel ensemble method with dynamic weights based on fitting (DWF) to solve the gas discrimination problem, regardless of the gas concentration, with high accuracy over extended periods of time. The DWF method uses a dynamic weighted combination of support vector machine (SVM) classifiers trained by the datasets that are collected at different time periods. In the testing of future datasets, the classifier weights are predicted by fitting functions, which are obtained by the proper fitting of the optimal weights during training. We compare the performance of the DWF method with that of competing methods in an experiment based on a public dataset that was compiled over a period of three years. The experimental results demonstrate that the DWF method outperforms the other methods considered. Furthermore, the DWF method can be further optimized by applying a fitting function that more closely matches the variation of the optimal weight over time.

## 1. Introduction

Electronic noses, a collection of broadly cross-reactive sensors connected to electronics and an effective pattern recognition system, are used to detect, classify and, where necessary, quantify a variety of chemical analytes or odors of concern in the area of interest [1]. The key issue in construction of such systems is the selection and stability of sensors. A phenomenon known as sensor drift has been recognized as one of the most serious impairments of the above performance [2].

Romain and co-workers systematically analyzed sensor drift [3]. They utilized a very comprehensive dataset, collected over a period of three years under real operating conditions [4], to provide further insight into the sensor drift problem with regard to both first- and second-order drift. In this paper, we focus exclusively on the first-order drift (hereafter referred to as “drift”) of the metal oxide sensor.

There are several different ways of drift reduction, which can be classified into three main categories. The first is the search for new materials that can reversibly interact with the relevant gas, so that the detected molecules unbind from the sensor material as soon as the gas has been purged from the sensor surface [5,6]. The second is the dynamical characterization of the sensor response. Some solutions based on periodically changing the working temperature of the sensor [7,8] have been implemented to minimize the effects of irreversibility in the sensor response due to poisoning. Additionally, the third is the use of sensor arrays and appropriate signal processing techniques, including feature extraction and pattern recognition techniques.

Note that the research of this paper is based on an assumption that all sensors function correctly. Sensor failure, which is another type of sensor degradation, has also attracted great attention. Pattern recognition techniques are also used to detect faults, such as [9–11].

This paper focuses on the gas discrimination using metal oxide gas sensor array, regardless of the gas concentration. Under the condition of other unchanged factors, such as sensor materials and number, environment, feature extraction methods, *etc.*, the influence of the sensor drift on discrimination is avoided or reduced over longer periods of time, just by improving the classification method. A classifier ensemble method with dynamic weights based on fitting (DWF) is proposed in this paper. Experimental results indicate that the performance of the DWF degrades more slowly over time than that of the static classifier ensembles. The DWF can mitigate the drift effect in metal oxide gas sensors for a longer period of time, thereby prolonging the lifetime of metal oxide gas sensors.

In the remainder of this paper, we first survey the existing work by the chemical sensing community on the problem of using classifier methods (Section 2). Next, the DWF method proposed in this paper is described (Section 3), and this is followed by a detailed description of the experiment (Section 4). Finally, the conclusions drawn from the results presented in this paper are presented (Section 5).

## 2. Related Work

In the early days, analytes were identified by a single classifier model, such as support vector machine (SVM), artificial neural network (ANN) and their derivatives. Lee *et al.* used a multi-layer neural network with an error back propagation learning algorithm as a gas pattern recognizer [12]. Polikar *et al.* used a neural network classifier and employed the hill-climb search algorithm to maximize the performance [13]. The authors in [14–17] also used ANN to identify the analytes of interest. Xu *et al.* used a Fuzzy ARTMAPclassifier, which is a constructive neural network model developed upon adaptive resonance theory (ART) and fuzzy set theory [18]. Some other researchers used SVM to solve classification in E-nose signal processing, such as [19–21].

The ensemble-based method is becoming an increasingly important method in the chemical sensing research community. An ensemble-based method trains a base classifier on each batch of datasets, followed by the construction of an ensemble of the base classifiers that are used to predict the testing dataset. Compared to using a single classifier model for prediction, classifier ensemble methods have been found to improve the performance, provided that the base models are sufficiently accurate and diverse in their predictions [22].

The methods of integrating base classifiers can be divided into two categories: static classifier ensembles and dynamic classifier ensembles. In a static classifier ensemble, the weight of each base classifier is decided before the classification phase. In a dynamic classifier ensemble, the weight of each base classifier is decided dynamically during the classification phase. Gao *et al.* used an ensemble of multilayer perceptions (MLPs), which are feedforward artificial neural network models [23], and that of four base models (namely MLP, MVLR, QMVLRand SVM) [24] to predict, simultaneously, both the classes and concentrations of odors. Shi *et al.* proposed an ensemble of density models, KNN, ANN and SVM, for odor discrimination [25]. Vergara *et al.* used a static ensemble of multiple SVMs to cope with the problem of drift in chemical gas sensors [26]. Wang *et al.* also proposed a static ensemble of SVMs, which is similar to that of Vergara *et al.*, only instead of the weight assignment method [27]. Amini *et al.* used an ensemble of SVMs or MLPs on data from a single metal oxide gas sensor (SP3-AQ2, FIS Inc., Hyogo, Japan) operated at six different rectangular heating voltage pulses (temperature modulation), to identify analytes regardless of concentration [28]. The experimental result showed that the accuracies obtained with the ensembles of SVMs or MLPs were almost equal, if using an identical integrating method. Very recently, Kadri *et al.* proposed a dynamic ensemble method called dynamic weighting of base models (DWBM) just for concentration estimation of some indoor air pollutants [29].

The performance of all ensemble methods described above degrade over time due to drift. Predictably, the performance of future ensemble methods will also degrade inevitably, if drift still exist. In the next section, we describe a novel ensemble method with dynamic weights based on fitting (DWF) to achieve improved performance (or to minimize degradation) over time.

## 3. Dynamic Classifier Ensemble and Predictions

### 3.1. The Problem of Static Classifier Ensembles

Consider a classification problem with a set of features, *x*, as the inputs and a class label (a gas/analyte in our problem), *y*, as the output. At every time step, *t*, a batch of examples, *S_t_* = (*X_t_*, *Y_t_*) = {(*x*_1_, *y*_1_), …, (*x_m_t__*, *y_m_t__*)}, of size, m*_t_*, is received. A classifier model, *f_t_*(*x*), is trained on the dataset, *St.* If *S*_1–_*_T_* (namely, *S_t_*, *t* = 1, …, *T*) are training datasets, the classifier ensemble, *h_T_*(*x*), is a weighted combination of the classifiers trained on *S*_1–_*_T_*, respectively, *i.e.*, $h_{T}\;\left(x\right)=\sum_{i=1}^{T}\beta_{i}f_{i}\;\left(x\right)$, where {*β*_1_, …, *β_T_* } is the set of classifier weights. The ensemble method in its most general form is described in Algorithm 1. The remaining problem is how to estimate the optimal weights.

A common and intuitive method to estimate the weights is to assign weights to the classifiers according to their prediction performance on batch *S_T_*, e.g., [26]. Wang *et al.*[27] use the weight, *β_i_* = *MSE_r_* – *MSE_i_*, for each classifier, *f*_i_, where *MSE_i_* is the mean square error of classifier *f_i_* on *S_T_* and *MSE_r_* is the mean square error of a classifier predicting randomly.


**Algorithm 1** The classifier ensemble method in its most general form.
**Require:** Datasets *S_t_* = {(*x*_1_, *y*_1_), …, (*x_m_t__*, *y_m_t__*)}*, t* = 1, …, *T*. 1:**for**
*t* = 1, …, *T*
**do** 2: Train the classifier, *f_t_* on *S_t_*; 3: Estimate the weight, *β_t_*, of *f_t_* using dataset *S_T_* by the appropriate technique; 4:**end for** 5:Normalize the weights, {*β*_1_, …, *β_T_*};**Ensure:** A set of classifiers, {*f*_1_, …, *f_T_*}, and corresponding weights, {*β*_1_, …, *β_T_*}.


To evaluate the performance of the ensembles, which use the weights estimated by the existing methods, the following experiment is carried out. The data used in this experiment was gathered by Vergara *et al.* [26]. They used 16 screen-printed MOXgas sensors (TGS2600, TGS2602, TGS2610 and TGS2620, four of each type) commercialized and manufactured by Figaro Inc. The resulting dataset comprises 13,910 recordings of the 16-sensor array when exposed to six distinct pure gaseous substances, namely, ammonia, acetaldehyde, acetone, ethylene, ethanol and toluene, each dosed in a wide variety of concentration values, ranging from 5 to 1,000 ppmv They map the sensor array response into a 128-dimensional feature vector, which resulted from a combination of the eight features described in [30] × 16 sensors. The measurements collected over the 36-month period are combined to form 10 batches, such that the number of measurements is as uniformly distributed as possible.

**Figure 1.**
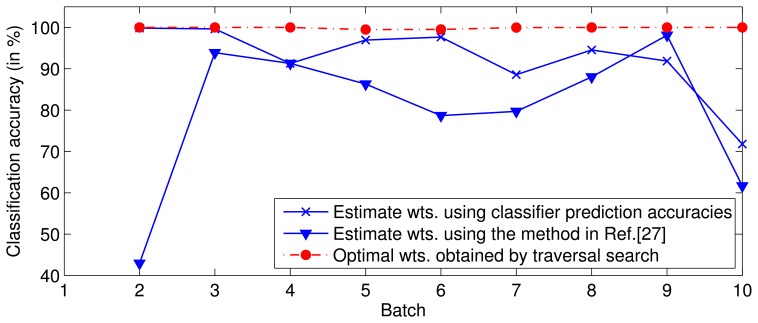
Performances of ensembles with classifier weights estimated by different methods.

In the experiment, for a given *T*, a set of classifiers (SVMs), *f*_1_, …, *f_T_*, are trained on batches *S*_1_, …, *S_T_*, respectively. Next, *S_T_*_+1_ is predicted by the ensemble of classifiers, *f*_1_, …, *f_T_*, with the optimal weights or the weights estimated by the above-mentioned methods. The optimal weights are obtained by a traversal search. The experimental result (Figure 1) indicates that the performance with the optimal weights is superior to that with the estimated weights, *i.e.*, the weights estimated by the existing methods are not optimal.

Most classifier ensemble methods, such as those described in references [26,27], are proposed based on an assumption that the distribution of the examples in the following batch, *S_T_*_+1_, does not change significantly from that in the current batch, *S_T_*. Thus, these methods use the examples in batch *S_T_* to estimate the weights, {*β*_1_, …, *β_T_* }, for *S_T_*_+1_. In other words, they use static weights for the following batches, *S_T_*_+1_, *S_T_*_+2_,…. For *S_T_*_+_*_n_*, if *n* is too large, *i.e.*, the time gap between time step *T* and *T* + *n* is too large, the relative drift may noticeably influence the prediction performance of the ensemble at *T* + *n*, of which the weights are estimated by some parameters of the ensemble at *T.*

**Figure 2.**
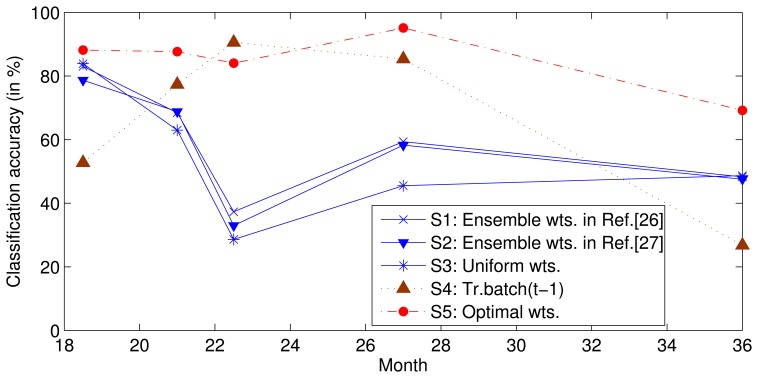
Performance of the classifiers under different settings.

To survey the influence of the relative drift, we used the same data and features as those used in a previous study [26] to complete the following experiment. We trained SVMs on batches *S*_1–5_ and then tested batches *S*_6–10_ using uniform weights (Setting 3) or a set of weights estimated using batch *S*_5_ with the methods proposed in [26,27] (Setting 1 and 2). To determine the best prediction performance of ensemble *f*_1–5_, we determined the traversal search sets of the optimal weights for each classifier in batches *S*_6–10_ (Setting 5); the ensemble classifier was found to perform with the greatest accuracy where the search range for each weight was [0,1] and the search step was 0.01, due to calculating time pressure. In addition, to verify the above assumption, we trained a classifier with data from only the previous batch and tested it on the current batch (Setting 4). The classification accuracies are shown in Figure 2, where the horizontal axis represents the mean time at which the data for each batch was collected. Note that the performance under Setting 4 is typically not the best. This non-ideal performance illustrates that the ensemble of the earlier SVMs with advisable weights can perform better than the SVM trained by only the previous batch. The optimal weights for each batch are obtained under Setting 5, and the performance is theoretically the best that can be obtained by class ensembles when {*X*_1–5_, *Y*_1–5_} and *X*_6–10_ are known and *Y*_6–10_ are unknown.

Predicting the optimal weight of each classifier for the incoming batch is difficult or impossible. The objective of this study is to predict the near-optimal weights. The performance under Settings 1 to 3 is close to the optimal performance (Setting 5) in the initial stages, but the performance degrades over time. This performance indicates that the near optimal weights can be obtained by using static weights, but the performance degrades with an increasing gap between the training dataset and test dataset. Because of this gap, a dynamic ensemble classifier is proposed in this paper to delay the degradation of performance and extend the life of the sensor.

### 3.2. Proposed Method

The performance of classifier ensembles with static weights degrades over time due to drift. To address this drift, a novel ensemble method with dynamic weights based on fitting (DWF), which is described below in its general form, is proposed in this paper to achieve improved performance (or to minimize degradation) over time.

We define *T* as the index of the current time step, and all sets of features, *X_t_*, and their class labels, *Y_t_*, in each batch, *S_t_*, *t* ≤ *T*, have been know, where *t* is the index of the time step. Thus, the dataset, *X_t_*, *t* ≤ *T*, can be predicted by the classifiers trained on not only the prior batch, *S_i_*, *i* < *t*, but also the later batch, *S_j_*, *j* > *t*. After training the classifiers on each batch, some classifiers, *f_t_*, *t* ≤ *T*, are received. All of the classifier ensembles are the weighted combinations of these classifiers.

At this point, the first important problem encountered is how to obtain the optimal or suboptimal weight of *f_t_* for each training batch. The experiment (Figure 1) has confirmed that the accuracies of the existing methods are much worse than the theoretical optimum accuracy. In this paper, we used a traversal search approach to determine the optimal weights of *f_i_*, *i* = 1, …, *T*, for each training batch, *S_j_*, *j* ≤ *T.* The search range for each weight is [0,1], and the search step is set to 0.05, due to the calculating time pressure. Table 1 lists the optimal weights of each classifier when *T* = 5. The use of the traversal search method can ensure that all of the weights are optimal, but the calculation requires a significant amount of time. The optimal weight matrix, namely, $\beta_{i}^{j}$ in the DWF method, consists of the weight of each classifier, *f_i_*, for training batch *S_j_*. The row vector of the matrix, $\beta_{i}^{j}$, is the subset of the optimal weights of the classifier at different time steps, and the column vector of that is the optimal weights assigned to all the classifiers at the corresponding time step. If scaling a column vector of $\beta_{i}^{j}$, the performance of the ensemble will stay invariable at the corresponding time step, because the proportion between the weights of the base classifiers do not change, which determines the performance, in fact. Thus, in the fitting stage, the curve of one classifier is fitted first. Then, the scaling factor of each column vector can be determined by the curve, *i.e.*, the 8th and 9th procedures in Algorithm 2-1.



**Algorithm 2** DWF method(2-1) The training and fitting stages.
**Require:** Training datasets, *S_t_* = {(*x*_1_, *y*_1_), …, (*x_m_t__*, *y_m_t__*)}, *t* = 1, …, *T*, and the mean measurement time, *w_t_*, of each dataset.1:**for**
*t* = 1, …, *T*
**do**2: Train a classifier, *f_t_*, on *S_t_*;3:**end for**4:**for**
*t* = 1, …, *T*
**do**5: Estimate the optimal weights, {
$\beta_{1}^{t}$,…
$\beta_{T}^{t}$}, of {*f*_1_, …, *f_T_*} for *S_t_* using the appropriate technique;6:**end for**7:Receive a *T* × *T* matrix, 
$\beta_{i}^{j}$;8:For a classifier, *i.e.*, *f*_*t*_0__, fit curve, *C*_*t*_0__(*w*), with {(
$\beta_{t_{0}}^{1}$, *ω*_1_), …, (
$\beta_{t_{0}}^{T}$, *ω_T_*)};9:Revise the t_0_th row vector, *β_t_*_0_, as [*C*_*t*_0__(*w*_1_), …, *C*_*t*_0__(*w_T_*)], by means of scaling each column vector of 
$\beta_{i}^{j}$;10:**for**
*t* = 1, …, *T* except *t*_0_
**do**11: Fit curve *C_t_*(*w*) with {(
$\beta_{t}^{1}$, *w*_1_), …, (
$\beta_{t}^{T}$, *w_T_*)};12:**end for****Ensure:** The classifiers, *f_t_*, and the corresponding fitting functions, *C_t_*(*w*)*, t* = 1, …, *T*.

(2-2) The testing stage.
**Require:** Test the dataset, *X_T_*_+_*_n_*, and the mean measurement time, *w_T_*_+_*_n_*, *n* > 0; the classifiers, *f_t_*, and the corresponding fitting functions, *C_t_*(*w*)*, t* = 1, …, *T*. 1:**for**
*t* = 1, …, *T*
**do** 2: Calculate the weight of *f_t_* at time *w_T_*_+_*_n_*, namely *C_t_*(*w_T_*_+_*_n_*); 3:**end for** 4:Test *X_T_*_+_*_n_* using the classifier ensemble, 
$h_{T+n}=\sum_{t=1}^{T}C_{t}\left(w_{T+n}\right)f_{t}$; 5:Estimate the labels: *Y_T_*_+_*_n_* = *h_T_*_+_*_n_*(*X_T_*_+_*_n_*);**Ensure:** Estimated labels *Y_T_*_+_*_n_*.


From $\beta_{i}^{j}$, the optimal weight of a classifier is observed to change over time. The weight of *f_i_* for a training batch, *S_j_*, and the corresponding mean measurement time of the batch form a two-dimensional array, ( $\beta_{i}^{j}$, *w_j_*). All of the arrays about *f_i_*, namely, {( $\beta_{i}^{1}$, *w*_1_), …, ( $\beta_{i}^{T}$, *w_T_*)}, can be used to fit a weight curve, *C_i_*(*w*), that is a function of time, *w*, for the classifier, *f_i_*, where *w_j_* is real time corresponding to index *j*. The weight of *f_i_* at time, *w_T_*_+_*_n_*, *n* = 1,2, …, can be predicted as *C_i_*(*w_T_*_+_*_n_*).

At this point, the second important problem encountered is determining what function can be used to fit these curves. Table 1 illustrates that, for most classifiers, the weight of *f_i_* is the maximum of all of the classifier weights at time step *i*, and the weight degrades from *i* to earlier or later. Thus, the fitting function, *C_i_*(*w*), should satisfy the following conditions: (I) *C_i_*(*w*) has maximum value at *w_i_* ; (II) *C_i_*(*w*_1_) – *C_i_*(*w*_2_) > 0 if *w*_2_ < *w*_1_ < *w_i_* or *w*_2_ > *w*_1_ > *w_i_*; (III) *C_i_*(*w*) > 0. Thus, the following function is proposed to fit the weight curve of the base classifier in this paper:

(1) $$y=\frac{c}{\exp\left(ax+b\right)+\exp\left(-\left(ax+b\right)\right)}+d$$

After fitting, four parameters (*a*, *b*, *c* and *d*) are determined for each function, *C_i_*(*w*), *i* = 1, …, *T*.

It should be noted that, scaling a column vector of $\beta_{i}^{j}$ does not change the accuracy of the classifier ensemble at the corresponding time step; however, it significantly affects the profile of each fit curve. Thus, a normalizing method is proposed for $\beta_{i}^{j}$ in the fitting stage. At first, a classifier, *i.e.*, *f*_*t*_0__, is chosen, and its curve, *C*_*t*_0__(*w*), is fitted by the *t*_0_th row vector of $\beta_{i}^{j}$ and the corresponding time, namely, {( $\beta_{t_{0}}^{1}$, *w*_1_), …, ( $\beta_{t_{0}}^{T}$ *, w_T_*)}. Then, scale each column vector of $\beta_{i}^{j}$, such that its *t*_0_th row vector is [*C*_*t*_0__(*w*_1_), …, *C*_*t*_0__(*w_T_*)]. Table 2 shows the normalized $\beta_{i}^{j}$ that is more conforming to the characteristics of the fitting function.

In the test stage, if the test dataset is *X_T_*_+_*_n_*, *n* > 0, the weight of each classifier, *f_i_*, at time step *T* + *n* is *C_i_*(*w_T_*_+_*_n_*). Thus, the final classifier ensemble at time step (*T* + *n*) is a weighted combination of these classifiers, namely:

(2) $$h_{T+n}\left(x\right)=\sum_{i=1}^{T}C_{i}\;\left(w_{T+n}\right)\;f_{i}\;\left(x\right)$$

The predicted label set of *X_T_*_+_*_n_* is:

(3) $$Y_{T+n}=h_{T+n}\;\left(X_{T+n}\right)$$

## 4. Experimental Section

In all our experiments, we train the multi-class SVMs (one-*vs.*-one strategy) with the RBFkernel using the publicly available LibSVM software. The datasets used in Section 3.1 are also used in the experiments. Because the dataset for toluene is lacking for almost one year, we test only the remaining five analytes.

At least four fitting points are required to calculate the four parameters in the fitting function. Thus, the datasets used in the training stage must be no less than 4 batches and, preferably, many more batches. On the other hand, the DWF method is proposed to mitigate the drift effect for a longer period of time, so enough batches of datasets are expected in the testing stage. Considering only 10 batches of datasets can be used in the training and testing stages, four ways of partitioning datasets are set up to compare the performance of the DWF with that of recent methods.

**Figure 3.**
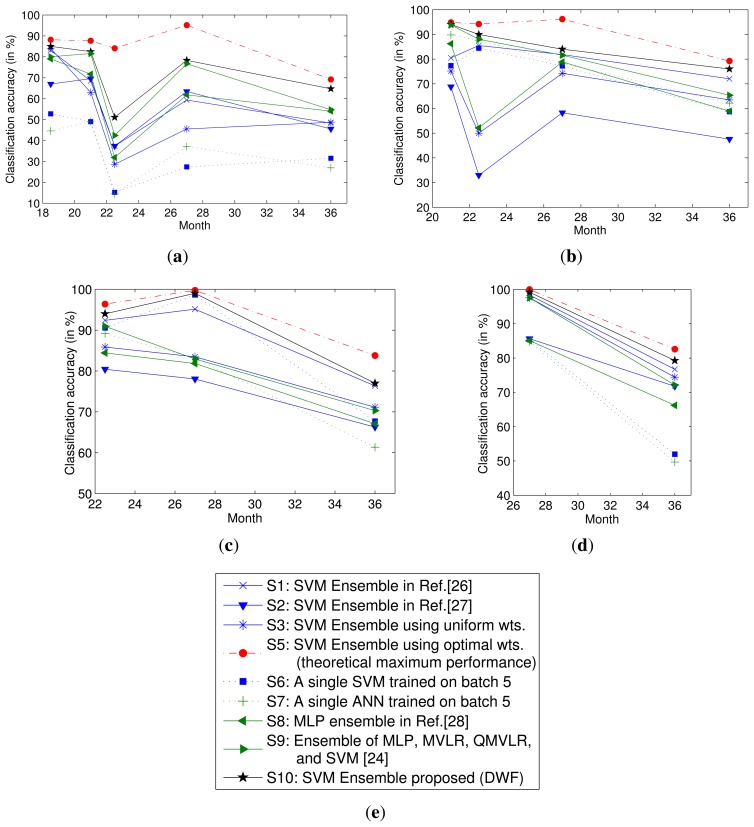
Performance of the classifiers under the proposed method and for other settings. (**a**)Training batches: *S*_1–5_ (collected from the first to the 16th month); testing batches: *S*_6–−10_ (collected from the 17th to the 36th month); (**b**) Training batches: *S*_1–6_ (collected from the first to the 20th month); testing batches: *S*_7–−10_ (collected from the 21st to the 36th month); (**c**) Training batches: *S*_1–7_ (collected from the first to the 21st month); testing batches: *S*_8–10_ (collected from the 22nd to the 36th month); (**d**) Training batches: *S*_1–8_ (collected from the first to the 23rd month); testing batches: *S*_9–10_ (collected from the 24th to the 36th month); (**e**) Legend.

The parameters of the five fitting functions are provided in Table 3, and the predicted weights of each classifier at time *t* ≥ 6 are provided in Table 4. The performance of the DWF method proposed in this paper is illustrated in Figure 3. For comparison, Figure 3 also illustrates the performance under other settings. Settings 1–3 are the same as those used in Figure 2. The theoretical maximum performance using the SVM ensemble is illustrated under Setting 5. Under Setting 6 and 7, an SVM and an ANN model are trained on the most recent training batch, respectively. Their performances are strong baselines, because the batch used in training is corrupted least by the drifted data from the past. Under Setting 8, an MLP ensemble [28] is used, and under Setting 9, an ensemble of four base models (namely MLP, MVLR, QMVLR and SVM) [24] is used. The performance of the DWF method is illustrated under Setting 10.

Experimental results show that the classifier using a single model depends heavily on selecting the training batch. In the classifier ensembles, the DWF method outperforms the other methods considered at all times. The ensemble under Setting 9 performs slightly worse than DWF. The performances under Settings 1, 2 and 8 are relatively close. Additionally, the performance under Setting 3 is worst at some time steps. All the performances degrade over time, but the performance of the DWF degrades more slowly over time than that of others. Therefore, it can be concluded that the DWF can mitigate the drift effect in metal oxide gas sensors for a longer period of time, thereby prolonging the lifetime of metal oxide gas sensors.

**Figure 4.**
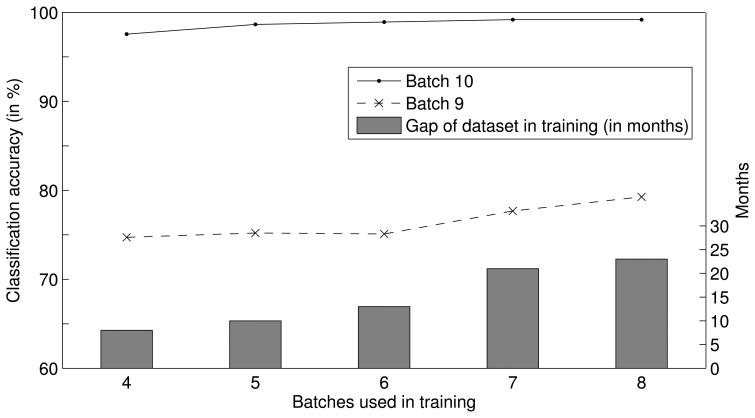
Performance of each ensemble using different gaps of the training dataset.

In the DWF approach, the weights in the test step are predicted by fitting functions, and the time span of the training dataset can affect the fitting result. It follows that the time span of the training dataset can affect the performance of the DWF approach. To verify this inference, we analyzed the impact of the time span on the classification accuracy in a set of experiments. We trained sets of SVMs using the datasets of different time spans for batches *S*_5–8_, *S*_4–8_, *S*_3–8_, *S*_2–8_, and *S*_1–8_, and combined five classifier ensembles, which are composed of four, five, six, seven and eight SVMs, respectively. Then, based on the DWF method, we predicted the weight of each SVM at the times of batches *S*_9_ and *S*_10_ and tested these predictions. Figure 4 illustrates the performance of each ensemble, where the histogram presents the time span of the training datasets of each ensemble, and the two profiles illustrate the classification accuracies of each ensemble tested on batches *S*_9_ and *S*_10_. The classification accuracies were found to increase as the time span of the training dataset increased, and the classification accuracy increased more substantially as the time gap between the training and test datasets increased. In conclusion, the performance of the DWF method can be improved by increasing the time span of the training dataset; in other words, the DWF approach can mitigate the drift effect for a longer period of time when the time span of the training dataset is increased.

## 5. Conclusions

This paper proposes a DWF method to mitigate the drift effect in metal oxide gas sensors. The experimental results indicate that the DWF method is able to cope well with sensor drift and perform better than the competing static-weighted ensemble methods. There are two vital problems in the DWF method. One problem involves the method for estimating the optimal weights in the training stage. For simplicity, the classifier prediction accuracy on recent training batches is commonly used to estimate the weights, but the experimental results confirm that the weights obtained by this method are not optimal. The performance of the ensemble assigning weights according to their prediction performances is much worse than that using weights obtained by the traversal search approach (Figure 1). However, the traversal search is too slow to be used in practice, so a novel solution is required to estimate the optimal weights. The other problem involves the selection of the fitting function. DWF relies on the proper selection of the fitting function. The curve features of the function should match the variation of the optimal weight over time. Besides, a fitting function with fewer parameters is expected. A further study will be performed to address these two problems.

## Figures and Tables

**Table 1.** Original optimal weights of each classifier used for the training datasets at different time steps, namely, $\beta_{i}^{j}$.

**Classifier/*f****_i_*	**Batch ID (The Mean Measurement Time in Months)/*j* (*w****_j_***)**				
	**1 (1.5)**	**2 (6.5)**	**3 (12)**	**4 (14.5)**	**5 (16)**
*f*_1_	0.50	0.05	0.05	0.15	0.35
*f*_2_	0.10	0.55	0	0.05	0
*f*_3_	0.25	0.15	0.90	0.20	0
*f*_4_	0.15	0.10	0.05	0.50	0
*f*_5_	0	0.15	0	0.1	0.65

**Table 2.** Normalized optimal weights of each classifier used for the training datasets at different time steps.

**Classifier/*f****_i_*	**Batch ID (The Mean Measurement Time in Months)/*j*** (*w_j_*)				
	**1 (1.5)**	**2 (6.5)**	**3(12)**	**4 (14.5)**	**5(16)**
*f*_1_	0.5001	0.1500	0.1500	0.1500	0.1500
*f*_2_	0.1000	1.6505	0	0.0500	0
*f*_3_	0.2500	0.4501	2.6993	0.1999	0
*f*_4_	0.1500	0.3001	0.1500	0.4999	0
*f*_5_	0	0.4501	0	0.1000	0.2785

**Table 3.** Parameters of the five fitting functions.

**Classifier/*f****_i_*	**Parameters of the Fitting Function**			
	**a**	**b**	**c**	**d**
*f*_1_	5.6821	3.0462	−8.0483	0.1500
*f*_2_	−0.9910	−0.4797	6.7208	0.8296
*f*_3_	−12.3862	−4.4880	68.5792	1.0641
*f*_4_	21.1944	−0.01274	0.1071	−20.9216
*f*_5_	−21.5056	1.3284	3.3586	0.2065

**Table 4.** Predicted weights of each classifier.

**Classifier/*f****_i_*	**Tested Batch ID (The Mean Measurement Time in Months)**/***j*** (***w****_j_*)				
	**6 (18.5)**	**7 (21)**	**8 (22.5)**	**9 (27)**	**10 (36)**
*f*_1_	0.0707	0.0687	0.0697	0.0774	0.1173
*f*_2_	0.2839	0.3481	0.3697	0.4260	0.6489
*f*_3_	0.5015	0.4872	0.4944	0.5489	0.8324
*f*_4_	0.0466	0.0014	−0.0298	−0.1588	−0.7601
*f*_5_	0.0973	0.0946	0.0960	0.1065	0.1616
